# Population pharmacokinetic study of the effect of polymorphisms in the ABCB1 and CES1 genes on the pharmacokinetics of dabigatran

**DOI:** 10.3389/fphar.2024.1454612

**Published:** 2024-11-15

**Authors:** Zhuan Yang, Wen Rui Tan, Qin Li, Ying Wang, Shijing Liu, Lu Chen, Yan Zhou, Chen Zeng, Yan Zeng, Yun Xiong, Qian Zhang, Na Li, Peng Du, Lin Liu, Jiyu Chen, Yan He

**Affiliations:** ^1^ Clinical Trials Center, The Affiliated Hospital of Guizhou Medical University, Guiyang, China; ^2^ School of Pharmacy, Guizhou Medical University, Guiyang, China; ^3^ Department of Pharmacology and System Physiology, University of Cincinnati College of Medicine, Cincinnati, OH, United States

**Keywords:** population pharmacokinetic, food effects, dabigatran, genetic polymorphism, ABCB1, CES1

## Abstract

**Purpose:**

The impact of genetic polymorphisms in the ABCB1 and CES1 genes on dabigatran plasma concentrations remains a subject of debate, and the purpose of this study was to quantitatively assess the effects of genetic polymorphisms on dabigatran esters in healthy Chinese subjects employed a population pharmacokinetic (PopPK) approach.

**Methods:**

In total, 1,926 pharmacokinetic (PK) samples from 123 healthy individuals who were given 150 mg of dabigatran orally during a fasting state or postprandially were analyzed using the PopPK model. A two-compartment model with first-order absorption was found to adequately describe the PK data.

**Results:**

The results showed that covariates food intake and ABCB1 SNP rs4148738 were shown to have statistically significant impacts. Specifically, in postprandial administration increased lag time (ALAG) and clearance (CL) by 2.65% and 0.51%, respectively, and decreased absorption rate constant (KA) by 0.24%. Additionally, in subjects with CT genotype ABCB1 (rs4148738), the central ventricular volume of distribution (V_2_) was increased by 0.38%.

**Conclusion:**

In summary, the PopPK model developed in this study was robust and effectively characterized the pharmacokinetics of dabigatran in healthy Chinese adults, demonstrating that both food and ABCB1 genetic variation significantly influence the absorption and plasma concentration levels of dabigatran.

## 1 Introduction

Atrial fibrillation (AF) affects tens of millions of people worldwide and is the most common cardiac arrhythmia. Patients with AF are also at increased risk of stroke and systemic embolism (Guo et al., 2012). Dabigatran, an inhibitor of thrombin activity, is recommended for preventing embolic stroke in patients with non-valvular atrial fibrillation (NVAF) (Hayama et al., 2014; January et al., 2019). Compared to warfarin, dabigatran showed a lower risk of bleeding while maintaining comparable efficacy (Maddox et al., 2013).

Predictable pharmacokinetic profile, making it possible to give fixed doses of dabigatran without the need for routine coagulation dabigatran has a profile monitoring (Paul et al., 2015; Kim et al., 2022). However, it has been shown that the pharmacokinetic and pharmacodynamic responses of dabigatran vary widely between individuals (Paré et al., 2013; Dimatteo et al., 2016). Reportedly, the inter-individual coefficient of variation for dabigatran plasma concentrations in patients with AF was 51%–64%, while the intra-individual coefficient of variation was 32%–40% (Chan et al., 2015). Even among healthy volunteers, the coefficient of variation of pharmacokinetics (PK) between subjects was approximately 30% (Liu et al., 2022). Some studies indicate that genetic polymorphisms affect plasma levels of dabigatran (Paré et al., 2013; Kanuri and Kreutz, 2019; Ross and Paré, 2013). Dabigatran etexilate is a substrate for the intestinal efflux transporter protein P-glycoprotein (P-gp, encoded by the (ATP binding cassette subfamily B member 1) ABCB1 gene) (Ji et al., 2021). It is absorbed and converted to the active metabolite by carboxylesterase 1 (CES1, encoded by the CES1 gene) (Ji et al., 2021; Shi et al., 2016). Therefore, the absorption and metabolism of dabigatran etexilate may be affected by polymorphisms in the ABCB1 and CES1 genes. In addition, the expression of ABCB1 and CES1 vary markedly between tissues and gender might suggest to affect drug absorption and drug metabolism. ABCB1 is expressed in tissues mainly in the adrenal gland, gastrointestinal tract and kidney (Witta et al., 2023; Choudhuri and Klaassen, 2006). As well, CES1 is expressed in tissues mainly in the liver, gallbladder, and gastrointestinal tract (Di, 2019; Hosokawa, 2008).

In the Randomized Evaluation of Long-Term Anticoagulation Therapy (RE-LY) assay, the CES1 single nucleotide polymorphisms (SNPs) rs2244613 and rs8192935 as well as the ABCB1 SNP rs4148738 were associated with either trough or peak concentrations of dabigatran (Paré et al., 2013). The results of several studies have shown that the CES1 polymorphisms rs8192935 and rs2244613 affect dabigatran blood concentrations (Ji et al., 2021; Liu et al., 2021; Gu et al., 2018). Earlier studies also suggested that the presence of ABCB1 SNPs (rs4148738 and rs1045642) may affect the equilibrium peak concentration of dabigatran (Kryukov et al., 2017). Furthermore, the dabigatran ABCB1 polymorphisms rs4148738 and rs1045642 were first associated with an increased risk of major hemorrhagic events in a study in a Chinese population (Zhu et al., 2022). However, Ji et al. found there was no significant difference in dabigatran PK/PD in the variant genotypes of ABCB1 SNPs rs4148738 and rs1045642 (Ji et al., 2021). Consistently, recent studies have shown that polymorphisms in ABCB1 rs4148738 and CES1 rs2244613 do not affect dabigatran concentration (Liu et al., 2021). Hence, the effect of genetic polymorphisms in the ABCB1 and CES1 genes on blood concentrations of dabigatran is unclear.

Population pharmacokinetic (PopPK) modeling is widely employed to determine the pharmacokinetic parameters of a specific population and to delve into the factors (covariates) that contribute to the variability in pharmacokinetics (Sherwin et al., 2012). Currently, the influence of genetic polymorphisms in the ABCB1 and CES1 genes on dabigatran plasma concentrations is currently a subject of debate; the PopPK approach offers a valuable tool to evaluate this effect. Furthermore, existing dabigatran dosing regimens (75 mg/110 mg/150 mg, twice daily) may still lead to hemorrhagic or embolic events in specific populations (Connolly et al., 2009; Carmo et al., 2016), and the number of studies and sample sizes of the effects of genetic polymorphisms on dabigatran plasma concentrations in Chinese subjects is relatively small. If healthy subjects are used to analyze the effect of genetic polymorphisms on the pharmacokinetics of dabigatran, there are very few factors affecting the metabolism of dabigatran *in vivo*, which more accurately represents the effect of genetic polymorphisms. Thus, the aim of this study was to quantitatively analyze the effects of genetic polymorphisms on dabigatran etexilate in Chinese subjects using the PopPK method with relatively abundant PK data.

## 2 Methods

### 2.1 Analysis population

The data analyzed in this study came from samples from a dabigatran bioequivalence study in healthy Chinese subjects, including 29 females and 94 males, for a total of 1926 PK samples. For the PopPK analysis, only data from the reference formulation were used (Boehringer Ingelheim International GmbH, Germany). The inclusion criteria, typical for bioequivalence clinical trials, required participants to be healthy as confirmed by medical history and physical examination. The subjects ranged in age from 18 to 43 years, weighed between 45.2 and 82.0 kg, were 145.5–182.5 cm in height, and had a body mass index (BMI) of 19.2–25.9 kg/m^2^. Before conducting bioequivalence and pharmacokinetic studies, all participants gave their consent in writing. Helsinki declaration, Good Clinical Practices and the current National rules for the study was conducted in accordance with the clinical studies. An ethics committee of Guizhou Medical University’s affiliated hospital approved the study protocol (*Ethical batch number*: 2024037K). Our study was registered in *ClinicalTrial.org* (NCT06387407).

### 2.2 Study design and sampling

All subjects received a single dose of dabigatran etexilate (Pradaxa, 150 mg) in 240 mL of warm boiled water either in the fasted state or within 30 min after a standard high-fat meal. Venous blood was collected in the fasting group and the postprandial group at predose 0 h and 0.25 h, 0.5 h, 0.75 h, 1 h, 1.33 h (80 min), 1.67 h (100 min), 2 h, 2.5 h, 3 h, 3.5 h, 4 h, 5 h, 6 h, 8 h, 12 h, 24 h, 36 h, 48 h (19 points) after each cycle. Blood samples were collected approximately 4 mL at a time in pre-cooled EDTA-K2 anticoagulated blood collection tubes and centrifuged within 1 h of collection. We centrifuged blood samples at 4°C and 1700 g for 10 min. All samples were placed in a refrigerator at or below −60°C for 2 h after centrifugation for assay analysis.

### 2.3 Determination of plasma dabigatran concentrations

The total concentration of dabigatran in sub-center plasma was quantified by liquid-phase secondary mass spectrometry (HPLC-MS/MS) method (Jhang et al., 2020; Lin et al., 2021). The linear range of the total dabigatran plasma concentration assay was 1.000 ng/mL to 300.0 ng/mL, and the lowest limit of quantification was 1.000 ng/mL. PK parameter analysis: done using WinNonlin 8.2 or above software. The PK parameter set was used to calculate the pharmacokinetic parameters for each subject from the non-compartmental model, including C_max_, AUC_0-t_, AUC_0-∞_, T_max_, and t_1/2_. The arithmetic mean, standard deviation, coefficient of variation, median, quartiles, maximum, minimum and geometric mean of each parameter were also calculated. Blood concentration (C)-time (t) data analysis: individual and mean c-t curves and semilogarithmic c-t curves were plotted using the PK concentration set; arithmetic mean, standard deviation, median, maximum, minimum, and coefficients of variation of the blood concentrations at each time point were listed.

### 2.4 Genotype analysis

Approximately 50 μL of genomic DNA was extracted from blood using the Blood Genomic DNA Extraction Kit (TIANGEN BIOTECH (BEJING) CO., LTD.), and tested for DNA concentration and purity. Rapid Polymerase Chain Reaction (PCR) with PrimeSTAR^®^ Max DNA Polymerase (TaKaRa).

For ABCB1 rs4148738, the sequence of the forward primer was 5′CTG​CAA​GGA​GAT​TTA​ACC​CC 3′; and the sequence of the reverse primer was 5′AAG​ACA​CCT​CAA​ACT​TGG​CC 3′. For ABCB1 rs1045642, the sequence of the forward primer was 5′ACA​AGG​AGG​GTC​AGG​TGA​TC 3′; and the sequence of the reverse primer was 5′GAA​CTC​TTG​TTT​TCA​GCT​GC 3′. For CES1 rs2244613, the sequence of the forward primer was 5′GCC​CTG​TAT​TCT​TGG​TGT​TT 3′; and the sequence of the reverse primer was 5′AGG​ACT​TGC​CCA​AAT​CAT​AG 3′. For CES1 rs8192935, the sequence of the forward primer was 5′TTA​TGG​TTC​AAT​ACC​CAA​TG 3′; and the sequence of the reverse primer was 5′AGA​AGC​AGT​TAA​GCA​GGT​GA 3′.

PCR cycling conditions were as follows: genotyping of carriers of the CES1 gene polymorphism marker (rs8192935); the PCR reaction solution amplification program consisted of a 10-s incubation at 95°C, followed by denaturation at 98°C for 10 s, annealing at 53°C for 15 s, and extension at 72°C for a sustained period of 30 s, which was repeated for 35 cycles. Genotyping of carriers of CES1 rs2244613, ABCB1 gene polymorphic variant rs1045642, and rs4148738 was also performed; the PCR reaction solution was initially denatured at 95°C for 10 s, denaturation at 98°C for 10 s, annealing at 55°C for 10 s, and extension at 72°C for 30 s for 35 cycles.

### 2.5 Model building

The PopPK analyses for dabigatran etexilate were performed in NONMEM (version 7.5.1, icon development solution, Ellicott, Maryland, United States) software using nonlinear mixed effects modeling. Graphical visualization of NONMEM results and simulations was performed using R (version 4.3.2, R Foundation for Statistical Computing) software. The method employed was the First-Order Conditional Estimation with Interaction (FOCEI). A two-compartment model with first-order uptake was used to describe the PK of dabigatran etexilate. Covariates including age, weight, height, BMI, and genetic polymorphisms were incorporated in the analyses, and covariates were selected with forward selection and backward elimination. The final model robustness was evaluated via bootstrap analyses, and the results were compared with the observed data to assess the predictive performance of the model. Standard goodness-of-fit (GOF) plots, likelihood ratio tests, and visual predictive check (VPC) were also used to evaluate the model. The model evaluation was further supported by R and PsN (version 5.0.0) software packages. Supplementary tools included Pirana (version 23.1.1) and Xpose (version 4.7.1) for additional support and visualization.

In NONMEM modeling, an exponential model is usually used to describe Inter-Individual Variability (IIV). The exponential model is used to express the degree of variation in individual parameters, where the relationship between individual parameters (θ_i_) and typical parameters (θ_tv_) is as follows (Equation 1):

$$\theta_{i}=\theta_{\mathrm{tv}}*\exp^{\eta i}$$ (1)

where η_i_ is the individual’s random effect, which is usually assumed to follow a normal distribution with mean 0 and variance ω^2^, i.e., η _i_ ∼N (0, ω^2^).

Residual Variability is often characterized using a combine residual model. This approach allows for the integration of complex structures into the residual error terms, thereby providing a more accurate representation of variability observed in the data. The equations describing a residual variability are usually shown below (Equation 2):

$$Y_{\mathrm{ij}}=F_{\mathrm{ij}}*\left(1+\varepsilon_{\text{ij},2}\right)+\varepsilon_{\text{ij},1}$$ (2)

where Y_ij_ is the observed value of the i^th^ individual at the j^th^ time point and F_ij_ is the corresponding model predicted value, ε_ij,1_ and ε_ij,2_ are additive and proportional intra-individual variants, consistent with a mean of 0 and variances of σ_1_^2^ and σ_2_^2^, respectively.

### 2.6 Covariate selection and model evaluation

After the base model was established, individual parameter values were analyzed for correlation with each covariate. Covariates that potentially had an effect on the base model parameters were initially screened based on statistical significance and biological plausibility. The stepwise covariate modeling (SCM) method was employed to assess the impact of demographic and genetic covariates on the pharmacokinetics (PK) of Dabigatran. For this method, the critical value of objective function value (OFV) for forward inclusion was set at 3.84 (P < 0.05), and that for backward elimination was set at 10.83 (P < 0.001). Covariates with significant influences were forward included and backward eliminated one by one to determine the final covariates to be included. The covariates examined in this study included age, height, weight, BMI, sex, diet, ABCB1 and CES1 genotypes of the subjects. But the way in which a covariate is introduced into the model depends on the type of data for that covariate, with continuous covariates being represented by exponential modeling formulas (Equation 3), and categorical covariates being represented by piece-wise modeling formulas (Equation 4).

$$\theta_{i}=\theta_{\mathrm{tv}}*\exp^{\eta i}*{\theta_{\mathrm{cov}}}^{\frac{{\mathrm{cov}}_{i}}{{\mathrm{cov}}_{\mathrm{median}}}}$$ (3)

$$\theta_{i}=\theta_{\mathrm{tv}}*\left(1+\text{COV}*\theta_{\mathrm{COV}}\right)$$ (4)

where θ_i_ represents the parameter of the i^th^ individual, θ_tv_ represents the typical value of the parameter, θ_cov_ represents the typical value of the covariate parameters, and η_i_ represents random variables with a mean of zero and a variance of ω^2^. COV_i_ is a continuous covariate, and COV_median_ is the median of continuous covariates. If there is a classification covariate, COV = 1; if there is no classification covariate, COV = 0. θ_cov_ is the correction parameter for the covariates to the model parameters.

## 3 Results

### 3.1 Demographic information

A total of 123 healthy subjects with a total of 1,926 blood concentration data were included in this study. The details of the healthy subjects are shown in Table 1. All healthy subjects, whether fasting (n = 61) or fed (n = 62), were administered a single dose of 150 mg of dabigatran etexilate. The median age, height, weight, and BMI of the subjects in the fasting group were 25 years, 165 cm, 58.8 kg, and 21.5 kg/m^2^, and the median age, height, weight, and BMI of the subjects in the postprandial group were 25 years, 164.8 cm, 59.7 kg, and 21.9 kg/m^2^, respectively.

**TABLE 1:** Demographic characteristics.

	Fasting group	Postprandial group
Male, n (%)	47 (38.21)	47 (38.21)
Female, n (%)	14 (11.38)	15 (12.20)
Age (years), mean (SD)	25.20 ± 4.17	24.94 ± 5.08
Weight, mean (SD)	58.82 ± 7.18	59.74 ± 7.71
Height, mean (SD)	165.2 ± 8.13	164.77 ± 8.02
BMI(kg/m^2^), mean (SD)	21.50 ± 1.57	21.95 ± 1.74
Trough concentration (ng/mL), mean (SD)	33.15 ± 12.06	33.91 ± 17.46
Peak concentration (ng/mL), mean (SD)	159.50 ± 57.28	114.89 ± 36.96

### 3.2 Distribution of genotypes

Four SNPs were genotyped in 99 healthy subjects receiving a single oral dose of 150 mg dabigatran etexilate capsules, and the genotype results are presented in Table 2.

**TABLE 2:** Allele frequencies by loci for the CES1 and ABCB1 in the subjects.

Gene	SNP	Genotype	N	Minor allele	MAF (%)
CES1	rs2244613	GG	40	T	36.87
		GT	45		
		TT	14		
	rs8192935	AA	49	G	26.77
		AG	47		
		GG	3		
ABCB1	rs4148738	CC	20	C	44.44
		CT	48		
		TT	31		
	rs1045642	AA	15	A	39.29
		AG	47		
		GG	36		

SNP: single-nucleotide polymorphism; MAF: minor allele frequency.

Among the 99 healthy subjects who received a single oral dose of 150 mg dabigatran etexilate capsules, genotyping revealed the following distribution for CES1 SNP rs2244613: 40 subjects had the GG genotype, 14 subjects had the TT genotype, and 45 subjects were heterozygous (GT). The minor allele (T) frequency for CES1 SNP rs2244613 was 36.87%. For CES1 SNP rs8192935, 49 subjects had the AA genotype, three subjects had the GG genotype, and 47 subjects were heterozygous (AG). The minor allele (G) frequency for CES1 SNP rs8192935 was 26.77%.

Additionally, there were 20 carriers of the CC genotype of ABCB1 SNP rs4148738, 31 homozygous carriers of the T allele, and 48 heterozygous carriers of one C allele; the frequency of the minor allele (C) of ABCB1 SNP rs4148738 was 44.44%. Fifteen subjects carried the AA genotype of ABCB1 SNP rs1045642, 36 subjects were G-allele homozygotes, and 47 patients were heterozygotes carrying one A allele; the frequency of the minor allele (A) of ABCB1 SNP rs1045642 was 39.29%.

### 3.3 Pharmacokinetics

**FIGURE 1:**
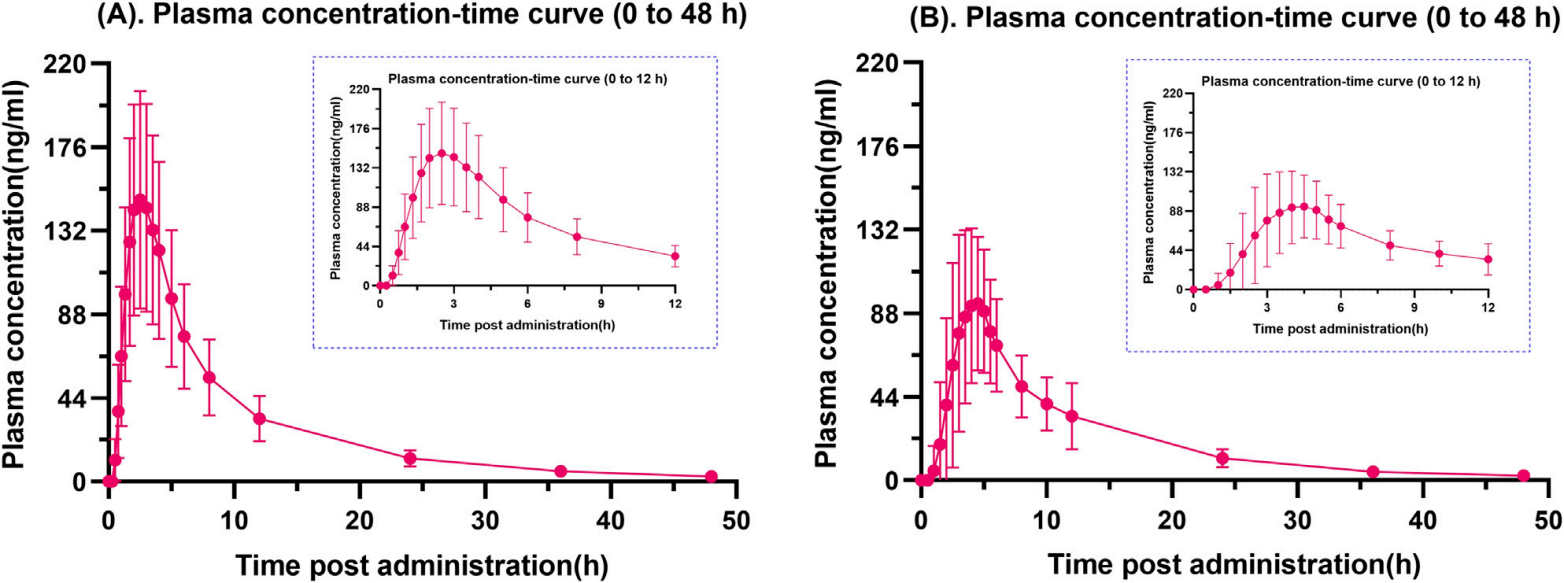
Fasting **(A)** and postprandial **(B)** plasma total dabigatran concentration-time profiles. A graphical depiction comparing the average plasma concentration of dabigatran over time in healthy Chinese individuals under fasting conditions **(A)** and after meals **(B)**. Every error bar represents the SD.

Mean plasma drug concentration-time curves for fasting and postprandial total dabigatran are shown in Figure 1. It is evident from the graph that the peak blood concentrations after the subjects took the standard high-fat meal were significantly lower than those taken on a fasting basis.

The results of the pharmacokinetic parameters analyzed by food effects, gender, and polymorphisms of the non-compartmental analysis are shown in Table 3. The peak time for a single oral dose of dabigatran etexilate capsules in healthy Chinese adults was about 2.4 h on an empty stomach and 4 h after a meal. Both scenarios showed a trend of linear kinetic characteristics. The mean AUC values were significantly higher when administered in the fasting state than in the postprandial state. Moreover, female subjects had slightly higher mean C_max_ and AUC values than male subjects. TT gene carriers of CES1 SNP rs2244613 had higher mean C_max_ and AUC values than GG and GT gene-carrying subjects. GG gene carriers of CES1 SNP rs8192935 had significantly higher mean C_max_ and AUC values than AA and AG gene carriers. In contrast, the ABCB1 SNPs (rs414873 and rs1045642) had no significant effect on C_max_ and AUC.

**TABLE 3:** Results of non-compartmental analysis of plasma dabigatran.

	N	C_max_ (ng/mL)	T_max_(h)	AUC_0-t_ (ng⋅h/mL)	AUC_0-∞_(ng⋅h/mL)	t_1/2_(h)
Food effect (N = 123)						
Fasting	61	159.56 ± 57.25	2.39 ± 0.60	1352.14 ± 454.75	1395.65 ± 466.86	10.07 ± 2.33
Postprandial	62	114.89 ± 36.96	4.44 ± 2.08	1049.14 ± 313.73	1071.37 ± 313.71	9.24 ± 1.32
SEX (N = 123)						
Male	94	136.33 ± 52.73	3.11 ± 1.09	1177.60 ± 435.79	1218.66 ± 446.37	9.79 ± 2.04
Female	29	139.36 ± 54.32	4.45 ± 3.07	1270.09 ± 347.61	1299.67 ± 361.98	9.19 ± 1.48
CES1 SNP rs2244613 (N = 99)						
GG	40	135.32 ± 62.60	3.63 ± 2.35	1187.89 ± 508.40	1216.80 ± 524.69	9.336 ± 1.90
GT	45	131.71 ± 49.90	3.55 ± 1.82	1176.47 ± 390.59	1216.03 ± 403.55	9.68 ± 1.79
TT	14	156.23 ± 52.73	2.76 ± 1.04	1286.87 ± 377.30	1322.27 ± 382.42	9.54 ± 1.20
CES1 SNP rs8192935 (N = 99)						
AA	49	134.43 ± 55.14	3.34 ± 2.06	1187.01 ± 464.69	1218.24 ± 478.55	9.59 ± 1.90
AG	47	134.20 ± 53.48	3.68 ± 1.93	1177.71 ± 403.16	1215.71 ± 414.47	9.50 ± 1.66
GG	3	211.03 ± 72.34	2.22 ± 0.69	1652.17 ± 382.85	1691.93 ± 389.58	9.17 ± 0.77
ABCB1 SNP rs4148738 (N = 99)						
CC	20	149.52 ± 45.69	3.40 ± 2.26	1312.07 ± 446.42	1321.88 ± 452.86	9.10 ± 1.46
CT	48	126.45 ± 58.26	3.73 ± 1.89	1145.77 ± 448.85	1192.18 ± 467.69	9.87 ± 2.15
TT	31	144.10 ± 56.54	3.11 ± 1.92	1201.09 ± 413.61	1236.92 ± 425.15	9.27 ± 1.01
ABCB1 SNP rs1045642 (N = 98)						
AA	15	145.85 ± 31.47	3.47 ± 2.50	1315.01 ± 374.43	1312.08 ± 372.97	9.22 ± 1.24
AG	47	126.51 ± 60.71	3.51 ± 1.54	1120.27 ± 466.94	1163.98 ± 480.75	9.63 ± 2.24
GG	36	145.82 ± 56.70	3.45 ± 2.30	1246.37 ± 421.31	1292.01 ± 438.79	9.54 ± 1.10

C_max_: the peak plasma concentration; T_max_: time to peak drug concentration; AUC_0-t_: area under the plasma concentration-time curve from time 0 to last time of quantifiable concentration; AUC_0-∞_: area under the plasma concentration-time curve from time zero extrapolated to infinite time; t_1/2_: elimination half-life. Values are expressed as mean and standard deviation (SD).

### 3.4 Effect of gene polymorphisms on peak and trough concentrations

**FIGURE 2:**
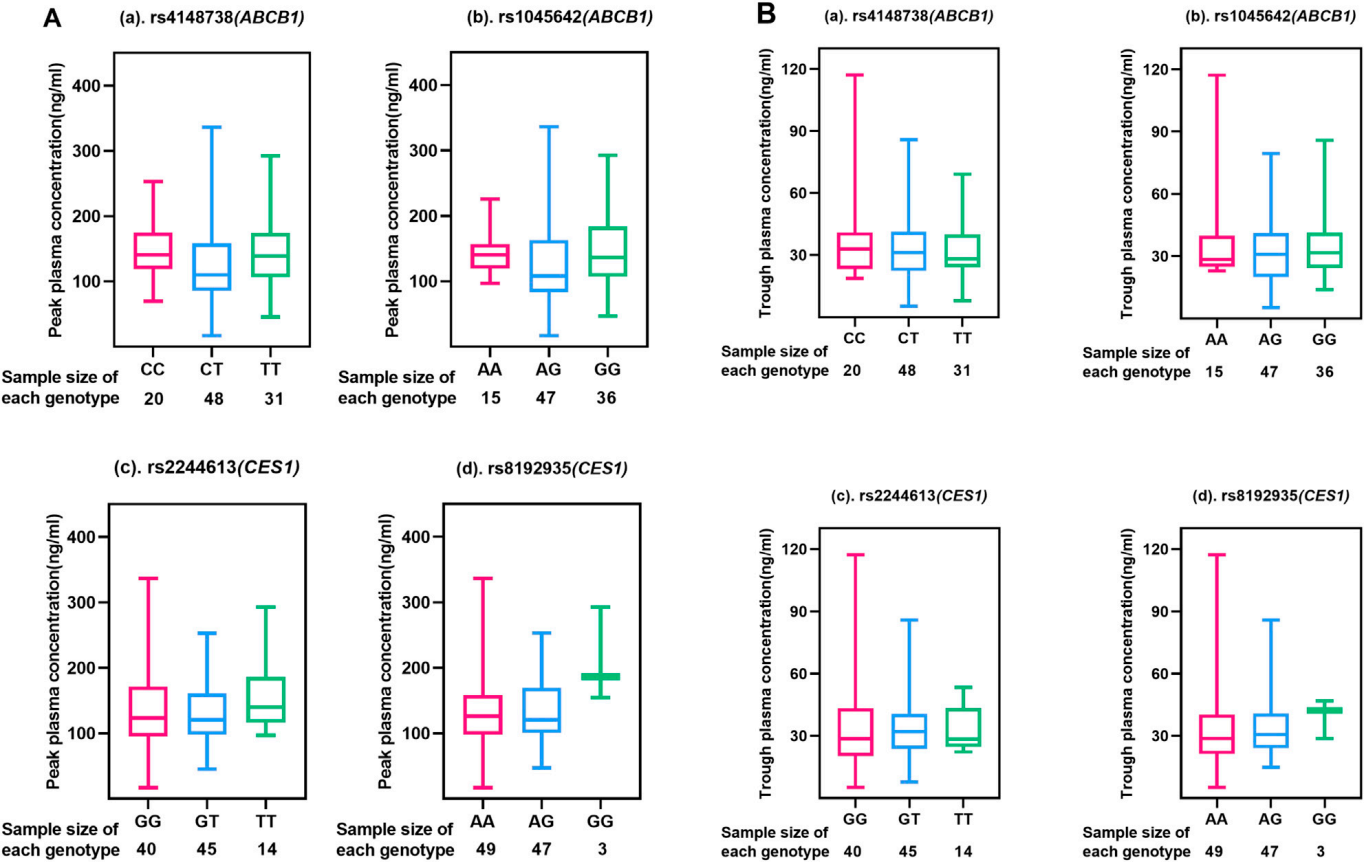
Peak and Trough **(A, B)** plasma concentrations of dabigatran in different genotypes of four SNPs. Each box-and-line plot describes the distribution of drug concentrations by showing medians, interquartile ranges (IQR), upper and lower whiskers, and outliers. (a) for ABCB1 SNP rs4148738 Genotype: CC (red), CT (blue), TT (green); Sample size: CC (20), CT (48), TT (31); (b) for ABCB1 SNP rs1045642 Genotype: AA (red), AG (blue), GG (green); Sample size: AA (15), AG (47), GG (36); (c) for CES1 SNP rs2244613 Genotype: GG (red), GT (blue), TT (green); Sample size: GG (40), GT (45), TT (14); (d) for CES1 SNP rs8192935 Genotype: AA (red), AG (blue), GG (green); Sample size: AA (49), AG (47), GG (3).

**FIGURE 3:**
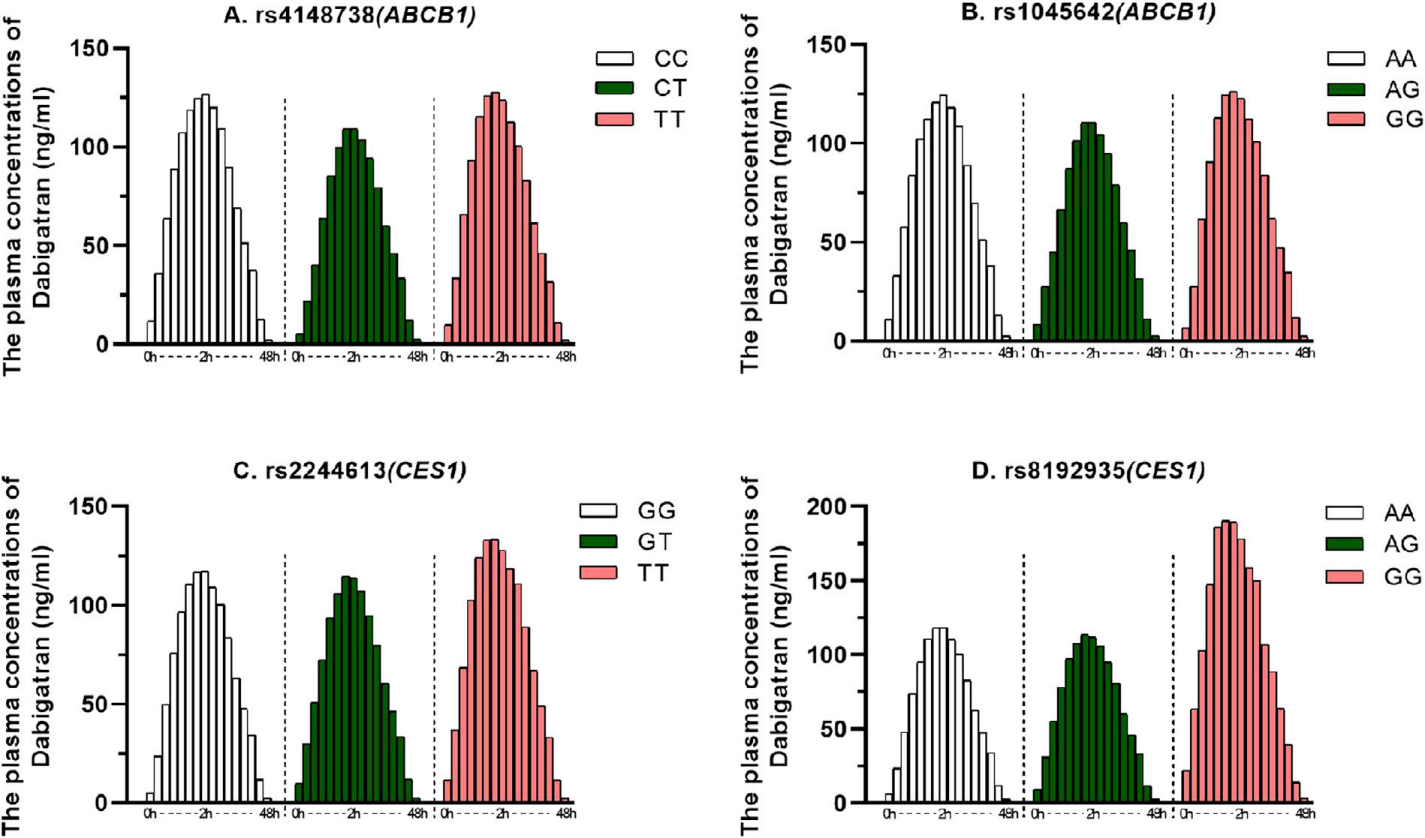
Dabigatran plasma concentrations for different genomic subpopulations. The horizontal coordinate indicates the sampling time after administration (0–48 h) and the vertical coordinate indicates the plasma concentration of dabigatran. **(A)** for ABCB1 SNP rs4148738 Genotype: CC (white, 20), CT (green, 48), TT (red, 31); **(B)**. for ABCB1 SNP rs1045642 Genotype: AA (white, 15), AG (green, 47), GG (red, 36); **(C)**. for CES1 SNP rs2244613 Genotype: GG (white, 40), GT (green, 45), TT (red, 14); **(D)**. for CES1 SNP rs8192935 Genotype: AA (white, 49), AG (green, 47), GG (red, 3).

A total of 123 subjects, 99 were genotyped for ABCB1 (rs4148738 and rs1045642) and CES1 (rs2244613 and rs8192935). We considered the concentration at 12 h after administration as the trough concentration of dabigatran in plasma (Zhu et al., 2022), the effects of genetic polymorphisms on dabigatran plasma peak and trough concentrations are shown in Figure 2. The effects of each of the four SNPs on peak and trough plasma concentrations of dabigatran, separately in males and females, are presented in Supplementary Figure 1. Statistical results showed no significant difference in peak plasma concentrations of dabigatran by sex for any of the four SNPs; however, differences were observed in trough plasma concentrations of dabigatran for rs4148738 (ABCB1) TT, rs2244613 (CES1) GT, and rs8192935 (CES1) AT genotypes. Additionally, Figure 3 displays histograms illustrating the plasma concentrations of dabigatran across different genomic subpopulations.

As can be seen in Figure 2A, no significant differences were seen among the scattered individual genotypes, indicating that the effect of genotype on peak drug concentration was not significant at these SNP loci, i.e., the peak drug concentration did not vary much among genotypes. In Figure 2B, different genotypes at different loci have significant effects on the median and range of plasma dabigatran trough concentrations. In rs4148738 (ABCB1), the CT genotype had a low median trough concentration of approximately 10 ng/mL with a narrow range. In rs1045642 (ABCB1), the GG genotype had a low median trough concentration of approximately 10 ng/mL and a small range of concentration variation. In rs2244613 (CES1), the median trough concentration of the TT genotype was low (10 ng/mL), the range was small, and the data were concentrated in the region of low concentration, but the sample size was small. In rs8192935 (CES1), AA had a median of approximately 20 ng/mL with a wide range of variability. ag had a slightly lower median and range of variability with a slightly narrower distribution of data. gg had the smallest sample size of three samples and the data were concentrated at lower concentrations of approximately 10 ng/mL. Different loci of the ABCB1 gene had a greater effect on grain concentration than the CES1 gene.

### 3.5 PK model

In analyzing the pharmacokinetic profile of dabigatran, one-, two-, and three-compartment compartment infrastructure models were examined separately. The two-compartment model was found to have a significantly lower OFV compared to the one-compartment model with a better fit and stable estimation, while the three-compartment model did not show a significant improvement in OFV compared to the two-compartment model, but was over-parameterized. The dabigatran PopPK model we developed was similarly chosen as a two-compartment model with absorption lag for first-level absorption, which allows for a better characterization of the data set, in line with previous studies modeling dabigatran ester concentrations (Liu et al., 2022; LIESENFELD et al., 2011). Comibine residual error models was used to describe intra-individual variation. Reference NONMEM control stream is furnished in the Supplementary Material.

The parameters and relative standard errors estimated by the final model are shown in Table 4. The typical values for the key pharmacokinetic parameters were as follows: central compartment clearance (CL) was 122 L/h, central volume of distribution (V_2_) was 245 L, intercompartment clearance (Q) was 70.80 L/h, peripheral volume of distribution (V_3_) was 756 L, absorption rate constant (Ka) was 0.38 h⁻^1^, and lag time (ALAG) was 0.48 h. The interindividual variation (IIV) for CL, V_2_, and ALAG was 12%, 8%, and 10%, respectively; and the model’s additive residual variance rate was 6.31 ng/mL.

**TABLE 4:** Final estimates of population PK parameters and bootstrap results.

Parameter	Final model		Bootstrap	
	Estimate (RSE%) [shr]	95%CI	Means	95% CI
KA(h^−1^)	0.38 (16.2%)	0.26∼0.50	0.34	0.28∼0.40
ALAG(h)	0.48 (5%)	0.44∼0.53	0.55	0.48∼0.63
CL(L/h^−1^)	122 (5.6%)	108.61∼135.39	125	115.23∼144.98
V_2_(L)	245 (14.1%)	177.18∼312.82	240	195.44∼276.26
Q(L/h^−1^)	70.80 (7.9%)	59.80∼81.80	65	55.31∼73.82
V_3_(L)	756 (10.6%)	599.20∼912.80	789	704.23∼954.93
Postprandial on KA	−0.24 (30%)	−0.39∼−0.10	−0.18	−0.27∼−0.03
Postprandial on ALAG	2.65 (8.7%)	2.20∼3.10	2.57	2.06∼2.79
Postprandial on CL	0.51 (25.5%)	0.26∼0.77	0.39	0.09∼0.51
rs4148738 on V_2_	0.38 (45.5%)	0.04∼0.71	0.33	0.04∼0.55
Exponential error	0.08 (24.9%)	0.04∼0.11	0.08	0.06∼0.10
Additive error	6.31 (14.7%)	4.50∼8.12	6.63	5.40∼8.08
Inter-individual variability (IIV)				
IIV_CL_	0.31 (12%) [7.2%]	−0.18∼0.79	0.27	0.15∼0.39
IIV_V2_	0.27 (8%) [0.1%]	−0.04∼0.58	0.34	0.26∼0.55
IIV ALAG	0.19 (10%) [14.6%]	−0.19∼0.57	0.15	0.08∼0.19
Residual variability	1 FIX [2.4%]	—	1 FIX	—

RSE: relative standard error; Shr: shrinkage (%), Bayesian shrinkage value.

KA: absorption rate constant; ALAG: lag time; CL: central chamber clearance; V_2_: central chamber distribution volume; Q: interventricular clearance; V_3_: peripheral chamber distribution volume: rs4148738: ABCB1 single nucleotide polymorphism (SNP).

During covariate analysis, it was found that food intake significantly influenced ALAG, CL, and Ka. Additionally, the CT genotype of ABCB1 SNP rs4148738 was found to affect the covariate of central compartment apparent volume of distribution (V_2_). Shrinkage of assessed between-individual random effects η is less than 15%, while the Bootstrap minimization success rate was 99.9%, and the median and 95% confidence intervals (CIs) from the bootstrap results closely matched the parameter estimates of the final model, further supporting its robustness.

**FIGURE 4:**
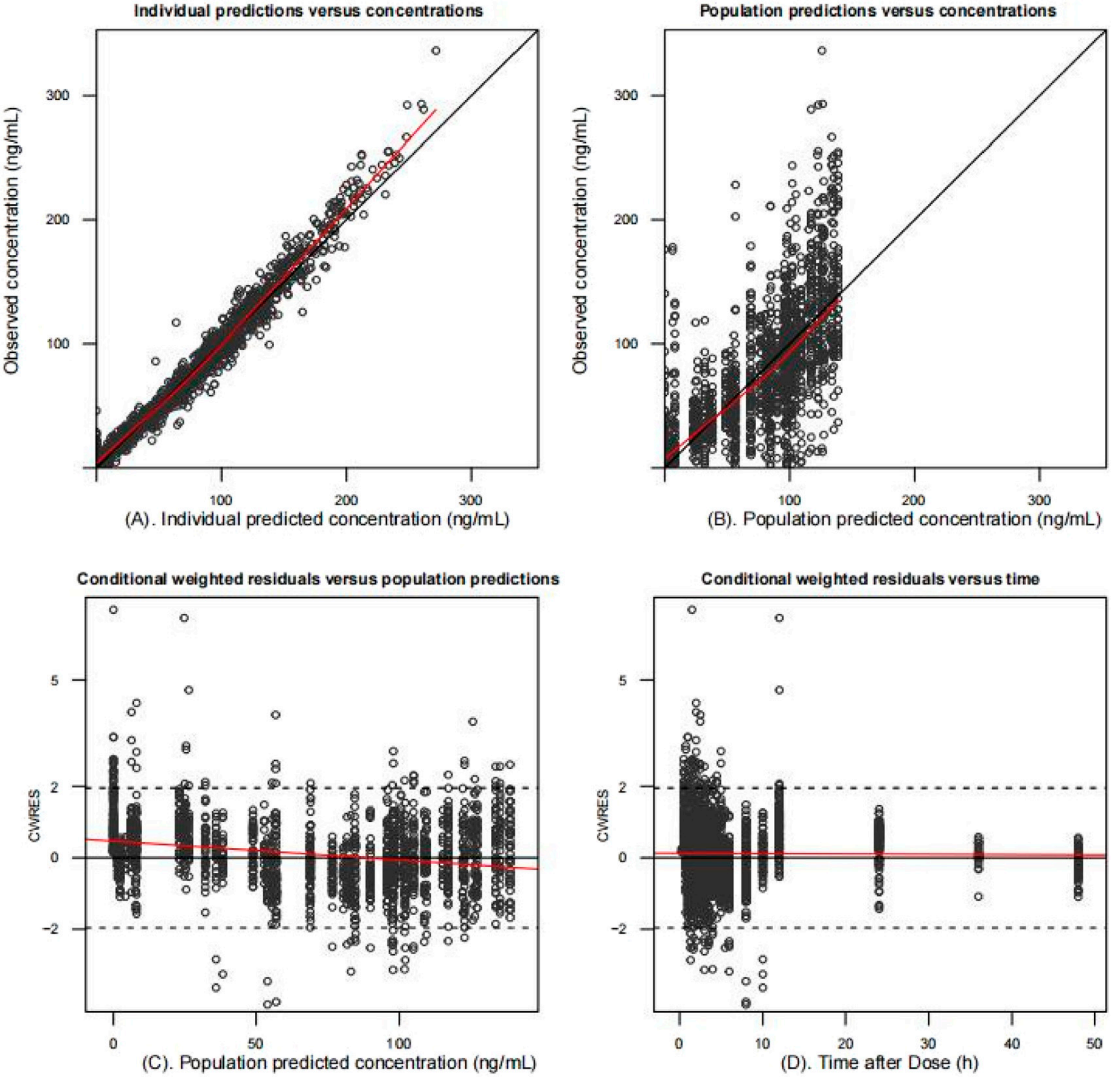
Goodness-of-fit plots of the final population pharmacokinetic model. Individual predicted values **(A)** and population predicted values **(B)** versus observed values. Conditionally weighted residuals vs. population predictions **(C)**; time vs. conditionally weighted residuals **(D)**. The black solid line is the reference line and the red solid line is the trend line. Most conditionally weighted residuals (CWRES) should be distributed between ± 2. The better the overlap between the reference and trend lines, the more accurate the model is.

**FIGURE 5:**
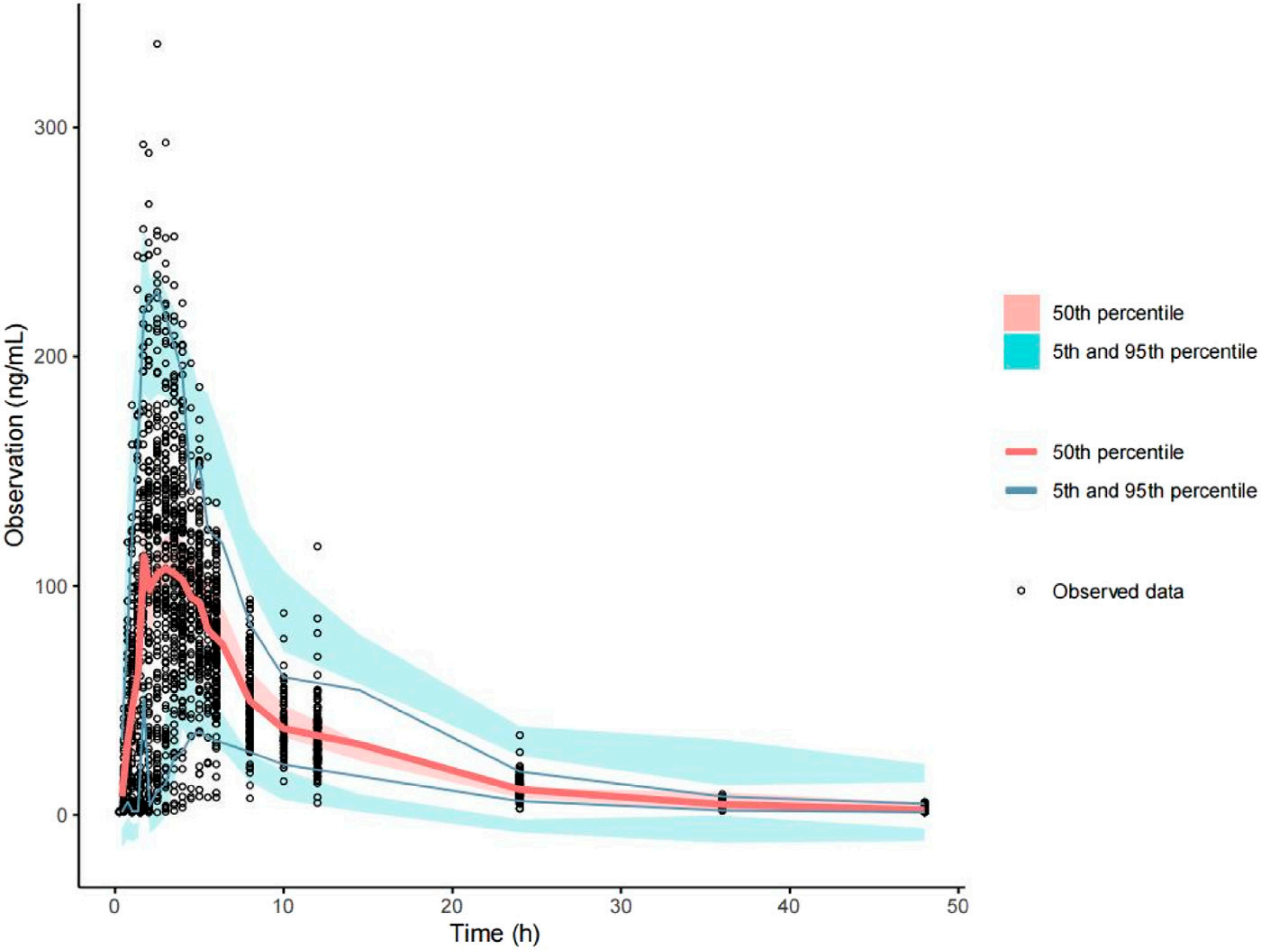
Predictive visual prediction check of the final model. Hollow dots represent observed concentrations (ng/mL) after administration. Solid lines represent the 5th, 50th, and 95th percentiles based on model simulation data. Red shading shows the 95% confidence interval for the median value predicted by the model, and the light blue shaded areas represent the 95% confidence intervals for the 5th and 95th percentiles predicted by the model.

The final model fit is demonstrated in Figure 4, where the goodness-of-fit plot illustrates a strong correlation between the population prediction (PRED) and individual prediction (IPRED) with the observed values. The data points are relatively evenly distributed around the diagonal line, and the PRED trend line nearly coincides with the diagonal line, indicating that the model fits the measured data well. Furthermore, the VPC plot, shown in Figure 5, reveals that the 95% CI of the model’s predicted values encompasses most of the observed data, suggesting that the model possesses good predictive power.

## 4 Discussions

NONMEM is a parametric population pharmacokinetic tool that allows for better parameter estimation and modeling of different subpopulations, helping us to more accurately understand the pharmacokinetic characteristics of these subpopulations. Furthermore, it enables us to more accurately depict the dynamics of drugs *in vivo* and to reveal the influence of genotype on pharmacokinetic parameters. This study is the first PopPK model developed using a relatively rich sample of dabigatran PK to specifically assess the correlation between alleles at different loci and dabigatran ester drug metabolism in healthy subjects. Our study focuses on PopPK modeling analyses using demographics, PK data, and genotype identification results from healthy subjects.

Previous studies have shown that factors affecting dabigatran plasma concentrations include renal function, gender, BMI, age, comorbidities, and genetic polymorphisms, with specific SNPs such as ABCB1 (rs1045642, rs4148738) and CES1 (rs2244613, rs8192935) having a greater impact on dabigatran pharmacokinetics (Thompson et al., 2023; Reilly et al., 2014). In this study, our data derived from healthy individuals excluded the effects of renal function and comorbid medications. The age range (18–43 years) and BMI range (19.2–25.9 kg/m^2^) were appropriate, and the smaller number of female subjects precluded a thorough analysis of gender effects on dabigatran pharmacokinetics. Consequently, the final model included covariates for food intake and one of the ABCB1 genotypes. Finally, the predicted values of the PK model are consistent with the observed dabigatran ester data, and the final model accurately and consistently characterizes the PopPK of dabigatran *in vivo*.

Consistent with published studies, the results of the blood concentration-time curves and non-compartmental modeling analyses, the mean AUC was approximately 8% higher in female subjects than in male subjects (Stangier et al., 2008; Zubiaur et al., 2020). It is clear that the median time to peak observed for postprandial administration was prolonged by approximately 2 h compared with fasting administration, which is consistent with the results of previous studies (Li et al., 2020). The studies have shown that the AUC of administration is similar in the two states, and that food does not affect the extent to which dabigatran is absorbed and can be taken with a meal (Li et al., 2020). However, the results of our study also showed that the fasting AUC values were about 28.86% higher than the postprandial AUC values, and the administration of high-fat meals could alter the AUC. In addition, postprandial administration was also shown in the final model to increase ALAG and CL by 2.65% and 0.51%, respectively, and decrease KA by 0.24%. It suggests that the absorption of the drug may be slowed or reduced in magnitude after administration of a high-fat diet, resulting in a reduction in peak concentrations; elimination of the drug is also less affected. Similar to our conclusion, the interaction between dabigatran and food has been conclusively demonstrated in rats (Shehab et al., 2019). Moreover, it has been suggested that the administration of dabigatran capsules on an empty stomach may increase the adverse effects of the drug on the digestive system, which may lead to therapeutic failure (Grześk et al., 2021). In patients treated with dabigatran, it is recommended to follow the regular dietary schedule and dietary components of the Cardiovascular Disease Prevention Guidelines (Grześk et al., 2021). Therefore, dabigatran-food interactions need to be carefully considered in the elderly, patients at high risk of bleeding, patients with reduced renal function, and individuals receiving complex medications (Antonijevic et al., 2017).

A total of 99 healthy Chinese subjects were genotyped in this study, and the genotype frequencies of the population we studied were similar to the genotype frequencies of similar previous studies (Liu et al., 2021). In our non-compartmental analysis, carriers of the GG gene for the CES1 SNP rs8192935 had significantly higher mean C_max_ and AUC values than carriers of the AA and AG genes, and this gene may influence plasma dabigatran concentrations. This finding is consistent with published studies of 202 Chinese patients (Ji et al., 2021) and 92 Caucasian patients (Dimatteo et al., 2016). In the RE-LY study, the CES1 SNP rs2244613 was associated with bleeding risk and trough concentrations of the drug (Paré et al., 2013). Polymorphisms in the ABCB1 SNPs (rs1045642 and rs4148738) and the CES1 SNP rs2244613 had no effect on dabigatran peak and trough concentrations in a study of 60 Caucasian patients with venous thromboembolism (Sychev et al., 2018). However, our results showed that TT gene carriers of CES1 SNP rs2244613 had higher mean C_max_ and AUC values than GG and GT gene-carrying subjects. The ABCB1 SNPs (rs414873 and rs1045642) had no significant effect on C_max_ and AUC. The results of the genetic polymorphisms on plasma dabigatran peak and trough concentrations in the present study showed that the peak drug concentrations did not differ significantly between genotypes, but different loci of the ABCB1 gene had a greater effect on the trough concentrations than the CES1 gene. Our final model also did not show an effect of the CES1 polymorphism, it may not affect plasma dabigatran concentrations, which is consistent with previous findings (Shi et al., 2016). However, given the existence of racial differences, CES1 polymorphisms may have different effects on plasma dabigatran in the Chinese population, except that we did not have enough sample size to derive the effect of CES1 polymorphisms. The size of the sample may influence the reliability of the data, particularly for genotypes with few subjects (only three for the GG type of rs8192935). Our model shows a 0.38% increase in the central volume of distribution (V_2_) in subjects with the CT genotype of ABCB1 SNP rs4148738. Genetic variation in the ABCB1 gene affects systemic concentrations of dabigatran (Wolking et al., 2015; Kanuri and Kreutz, 2019). Multiple findings suggest that an SNP (rs4148738) in the ABCB1 gene is associated with elevated plasma dabigatran peak concentration values (Paré et al., 2013; Kanuri and Kreutz, 2019; Sychev et al., 2018).

The limited number of studies on CES1 polymorphisms within the Chinese population, coupled with their relatively small sample sizes, underscores the need for further investigation into the effects of CES1 gene variations on dabigatran plasma concentrations. A more robust study involving a larger cohort is essential to validate these findings and understand their implications for dabigatran exposure *in vivo*. Patients undergoing treatment with dabigatran may encounter suboptimal therapeutic outcomes or severe adverse reactions. When standard clinical factors such as age, gender, liver and kidney function, and concurrent drug use fail to explain these discrepancies, the investigation into genetic factors becomes crucial. This study could provide a basis for exploring the relationship between genetic polymorphisms and dabigatran exposure *in vivo*.

## 5 Conclusion

Our study evaluated the population pharmacokinetics of dabigatran in healthy subjects with different genotypes and dietary status, and the final model describes the PK data for dabigatran well. The results of our final population pharmacokinetic modeling showed that dietary status had an effect on the absorption and elimination process of dabigatran, whereas ABCB1 SNP rs4148738 influenced the distribution process of dabigatran *in vivo*. Therefore, genotype identification and dietary status modification may be performed in patients at high risk of bleeding treated with dabigatran to improve drug safety and optimize therapeutic efficacy. This study may provide a basis for exploring the relationship between genetic polymorphisms and dietary status with dabigatran exposure *in vivo*.

## Data Availability

The original contributions presented in the study are included in the article/supplementary material, further inquiries can be directed to the corresponding author/s.
