# Supplementary material for: Population pharmacokinetic study of the effect of polymorphisms in the ABCB1 and CES1 genes on the pharmacokinetics of dabigatran
Source: Front Pharmacol. 2024 Nov 15;15:1454612. doi: 10.3389/fphar.2024.1454612 (PMC11605329; doi:10.3389/fphar.2024.1454612)
Supplement: Supplementary file 1 [file DataSheet1.docx]

Supplementary Material

# Supplementary Data

**NONMEM control stream of the final model**

$PROB FINAL MODEL

$INPUT ID TIME AMT DV MDV EVID CMT YS AGE SEX HT WT BMI ABCB1C ABCB1CT ABCB1T ABCB1A ABCB1AG ABCB1G CES1G CES1GT CES1TT CES1A CES1AG CES1G5 PT1 PTA1 APTT1 TT1 PT2 PTA2 APTT2 TT2 PT3 PTA3 APTT3 TT3

$DATA DBJQ-R_v3.csv IGNORE=@

$SUBROUTINE ADVAN6 TOL=6

$MODEL

COMP(COMP1, DEFDOSE)

COMP(COMP2, CENTRAL)

COMP(COMP3, PERIPHER)

$PK

;;; V2ABCB1CT-DEFINITION START

IF(ABCB1CT.EQ.0) V2ABCB1CT = 1 ;

IF(ABCB1CT.EQ.1) V2ABCB1CT = ( 1 + THETA(12))

;;; V2ABCB1CT-DEFINITION END

;;; V2-RELATION START

V2COV=V2ABCB1CT

;;; V2-RELATION END

;;; KAYS-DEFINITION START

IF(YS.EQ.0) KAYS = 1 ;

IF(YS.EQ.1) KAYS = ( 1 + THETA(11))

;;; KAYS-DEFINITION END

;;; KA-RELATION START

KACOV=KAYS

;;; KA-RELATION END

;;; CLYS-DEFINITION START

IF(YS.EQ.0) CLYS = 1 ;

IF(YS.EQ.1) CLYS = ( 1 + THETA(10))

;;; CLYS-DEFINITION END

;;; CL-RELATION START

CLCOV=CLYS

;;; CL-RELATION END

;;; ALAG1YS-DEFINITION START

IF(YS.EQ.0) ALAG1YS = 1 ;

IF(YS.EQ.1) ALAG1YS = ( 1 + THETA(9))

;;; ALAG1YS-DEFINITION END

;;; ALAG1-RELATION START

ALAG1COV=ALAG1YS

;;; ALAG1-RELATION END

TVKA=THETA(1)

TVKA = KACOV*TVKA

KA = TVKA

TVCL=THETA(2)

TVCL = CLCOV*TVCL

CL = TVCL* EXP(ETA(1))

TVV2=THETA(3)

TVV2 = V2COV*TVV2

V2 = TVV2 * EXP(ETA(2))

TVQ = THETA(4)

Q=TVQ

TVV3 = THETA(5)

V3=TVV3

TVALAG1=THETA(8)

TVALAG1 = ALAG1COV*TVALAG1

ALAG1=TVALAG1 * EXP(ETA(3))

S2 = V2/1000

$DES

K = CL/V2

K23 = Q/V2

K32 = Q/V3

DADT(1) = -KA*A(1)

DADT(2) = -K*A(2) -K23*A(2) +KA*A(1) +K32*A(3)

DADT(3) = -K32*A(3) +K23*A(2)

$THETA

(0,0.273295389265221) ; KA

(0,160.307067519302) ; CL

(0,191.015791394746) ; V2

(0,73.3937770233054) ; Q

(0,912.423926928567) ; V3

(0,0.0808644349923525) ; exponential error

(0,7.84235994018715) ; additive error

(0,1.05590514567162) ; ALAG1

$THETA (-1,0.997797165116948,5) ; ALAG1YS1

$THETA (-1,-0.235923213422901,5) ; CLYS1

$THETA (-1,0.227632154630835,5) ; KAYS1

$THETA (-1,0.287016882380804,5) ; V2ABCB1CT1

$OMEGA

0.25841998832159 ; IIV/BSV CL

0.435018578511045 ; IIV/BSV V2

0.139217136178069 ; IIV/BSV ALAG1

$SIGMA

1 FIX ; residual variability

$ERROR

IPRED = F

IRES = DV-IPRED

W = SQRT(THETA(6)**2*IPRED**2 + THETA(7)**2)

IF (W.EQ.0) W = 1

IWRES = IRES/W

Y= IPRED+W*ERR(1)

$ESTIMATION METHOD=1 INTERACTION MAXEVAL=9999 SIG=3 PRINT=5 NOABORT POSTHOC MCETA=1

$COVARIANCE PRINT=E UNCONDITIONAL

# Supplementary Figure 1


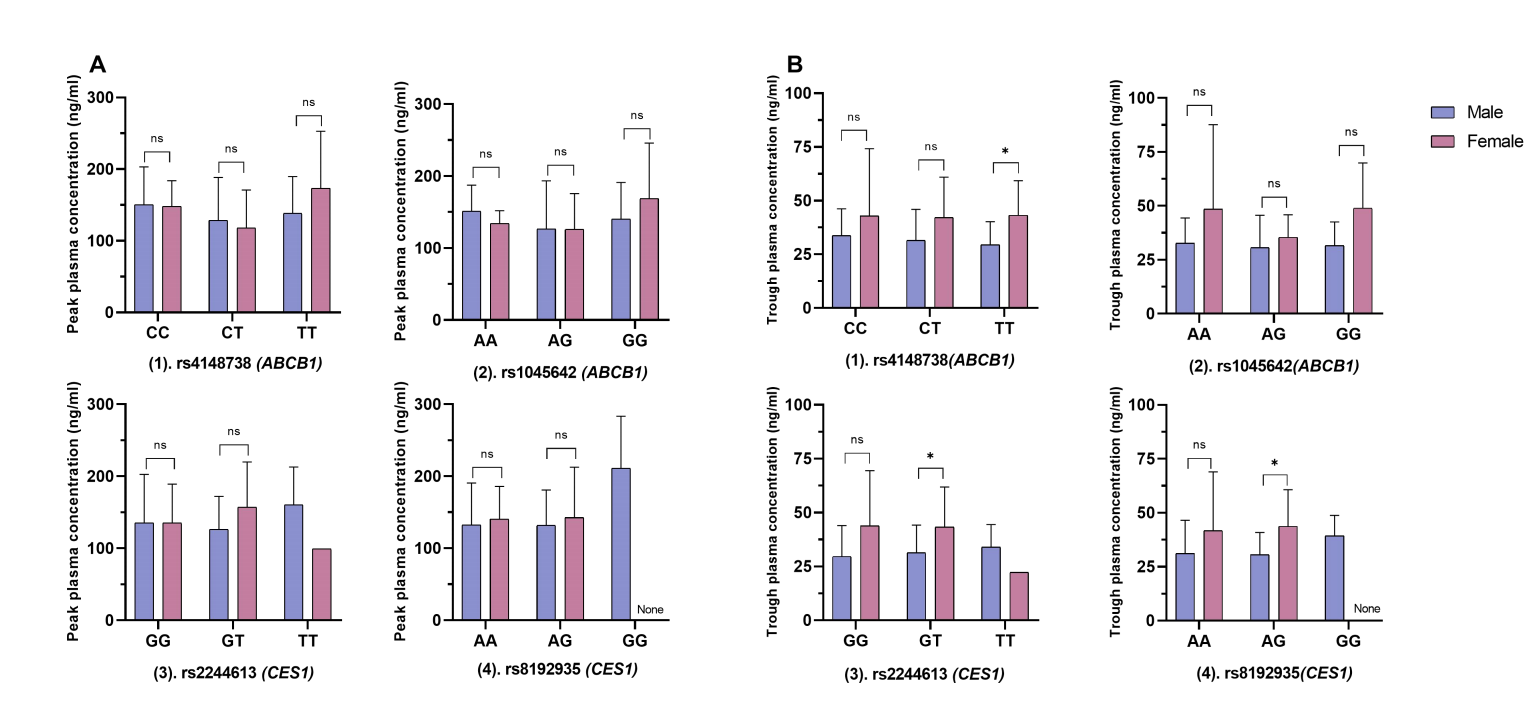


**Supplementary Figure 1.** The effects of each of the four SNPs on peak and trough plasma concentrations of dabigatran, separately in males and females.

The horizontal coordinates represent the genotypes corresponding to each SNP, while the vertical coordinates indicate either (A) peak or (B) trough plasma dabigatran concentrations. The purple color signifies males, and the red color signifies females. (1). for ABCB1 SNP rs4148738; (2). for ABCB1 SNP rs1045642; (3). for CES1 SNP rs2244613; (4). for CES1 SNP rs8192935.

.
